# Distinct behavioral traits and associated brain regions in mouse models for obsessive–compulsive disorder

**DOI:** 10.1186/s12993-021-00177-x

**Published:** 2021-05-18

**Authors:** Xiao Chen, Jihui Yue, Yuchong Luo, Lianyan Huang, Boxing Li, Shenglin Wen

**Affiliations:** 1 Department of Psychology, The Fifth Affiliated Hospital, Sun Yat-Sen University, No.52 Meihua West Road, Zhuhai, 519000 Guangdong Province China; 2 Neuroscience Program, Department of Physiology, Guangdong Provincial Key Laboratory of Brain Function and Disease, Zhongshan School of Medicine, Sun Yat-Sen University, Guangzhou, 510810 China; 3 Neuroscience Program, Department of Pathophysiology, Guangdong Provincial Key Laboratory of Brain Function and Disease, Zhongshan School of Medicine, Sun Yat-Sen University, Guangzhou, 510810 China

**Keywords:** Obsessive–compulsive disorder, Behavior, Brain area, Neuronal activity

## Abstract

**Background:**

Obsessive–compulsive disorder (OCD) is a mental disease with heterogeneous behavioral phenotypes, including repetitive behaviors, anxiety, and impairments in cognitive functions. The brain regions related to the behavioral heterogeneity, however, are unknown.

**Methods:**

We systematically examined the behavioral phenotypes of three OCD mouse models induced by pharmacological reagents [RU24969, 8-hydroxy-DPAT hydrobromide (8-OH-DPAT), and 1-(3-chlorophenyl) piperazine hydrochloride-99% (MCPP)], and compared the activated brain regions in each model, respectively.

**Results:**

We found that the mouse models presented distinct OCD-like behavioral traits. RU24969-treated mice exhibited repetitive circling, anxiety, and impairments in recognition memory. 8-OH-DPAT-treated mice exhibited excessive spray-induced grooming as well as impairments in recognition memory. MCPP-treated mice showed only excessive self-grooming. To determine the brain regions related to these distinct behavioral traits, we examined c-fos expression to indicate the neuronal activation in the brain. Our results showed that RU24969-treated mice exhibited increased c-fos expression in the orbitofrontal cortex (OFC), anterior cingulate cortex (ACC), prelimbic cortex (PrL), infralimbic cortex (IL), nucleus accumbens (NAc), hypothalamus, bed nucleus of the stria terminalis, lateral division, intermediate part (BSTLD), and interstitial nucleus of the posterior limb of the anterior commissure, lateral part (IPACL), whereas in 8-OH-DPAT-treated mice showed increased c-fos expression in the ACC, PrL, IL, OFC, NAc shell, and hypothalamus. By contrast, MCPP did not induce higher c-fos expression in the cortex than control groups.

**Conclusion:**

Our results indicate that different OCD mouse models exhibited distinct behavioral traits, which may be mediated by the activation of different brain regions.

**Supplementary Information:**

The online version contains supplementary material available at 10.1186/s12993-021-00177-x.

## Highlights

- Three mouse models of obsessive–compulsive disorder (OCD) were established.
- RU24969, 8-OH-DPAT, or MCPP administration caused specific OCD-like behaviors.
- Regional neuronal activity was assessed using c-fos expression levels.
- Expression of c-fos differed among the three newly established OCD mouse models.
- Distinct OCD symptoms may be treated by region-specific targeted therapy.

## Introduction

Obsessive–compulsive disorder (OCD) is a mental disorder mainly characterized by obsessive and compulsive behaviors. The prevalence of OCD is ~ 2.3% in the population [1], and most of the patients develop symptoms before 35 years old [2]. Selective serotonin reuptake inhibitors comprise the first-line treatment of OCD patients, but approximately half of the OCD patients fail to fully respond to this treatment [3]. Therefore, it is urgent to elucidate the mechanisms and causes of OCD.

OCD symptoms include obsessive (e.g., fear of contamination, the need to order things symmetrically, and aggressive, sexual, or religious thoughts) and compulsive (e.g., excessive washing, checking, ordering, counting, and repeating) traits [4]. Besides, OCD is a highly heterogeneous disease, with which many patients experience anxiety and cognitive deficits additionally. The symptoms vary widely among patients [5], and different dimensions of OCD symptoms may be caused by distinct neurobiological mechanisms [6]. Previous studies proposed that the dysfunction of parallel, partly segregated cortico–striato-thalamo-cortical (CSTC) loops, including serotoninergic, dopaminergic, and glutamatergic systems, are related to different cognitive-affective processes in OCD [4, 7–9]. For example, Mataix-Cols et al. used functional magnetic resonance imaging (fMRI) to measure neurological activity in patients with different symptom dimensions of OCD. They found that the activities of the bilateral ventromedial prefrontal regions and the right caudate nucleus were activated in patients with washing symptoms than in the control population.

Moreover, checking symptoms were accompanied by increased activity of the putamen/globus pallidus, thalamus, and dorsal cortical areas, whereas the activities of the left precentral gyrus and right orbitofrontal cortex in patients with hoarding symptoms were increased [6]. Therefore, each symptom may be mediated by relatively distinct brain regions or circuits. Targeted treatment on relevant brain regions may enable us to develop precise treatment, thereby improving treatment effectiveness in OCD.

Animal models are widely used to explore the physiological and pathological characteristics of OCD. However, the behavioral phenotypes of the OCD animal model were quite different among different models. Additionally, CSTC circuit anomalies were only detected in some models [10, 11]. The studies that systematically compare the behavioral traits and abnormal brain circuits between different animal models are entirely lacking.

In this study, we systematically examined the behavioral phenotypes of three OCD mouse models induced by pharmacological reagents (RU24969, 8-OH-DPAT, and MCPP), and compared the activated brain regions in each model, respectively. Our results showed that different OCD mouse models exhibited distinct behavioral traits, which may be mediated by the activation of different brain regions.

## Materials and methods

### Animals

All experiments were performed in accordance with the Guidelines for the Care and Use of Laboratory Animals by the National Institutes of Health, and the animal experiment protocols have been approved by the Institutional Animal Care and Use Committee of Sun Yat-Sen University. To avoid the impact of estrogen changes in the female mice on the behavior results, we only used male C57BJ6 mice (8–10 weeks old) in the study. 10–13 mice were used in each group in the behavioral experiments. Six mice in the 8-OH-DPAT group and MCPP group were used in the NOR test. In the cFos experiment, three mice were used in each group. Three brain slices with different cross-sections were selected from the same brain area in each mouse. All experimental mice were reared under standard laboratory conditions (12-h light–dark cycle, lights on at 21:00, food and water freely available, the temperature at 22 °C, humidity at 60%) in mouse cages covered with corncob litter. Mice were reared for at least 1 week to familiarize themselves with the environment before starting the formal experiments. Mice were assigned randomly to experimental groups.

The environmental factors such as sound, light, and injection stimulation can profoundly impact behavior results and c-fos expression. To minimize these influences, we treated each group under the same conditions, including consistent light intensity, a quiet testing room, and being handled in the same way for at least a week to ensure the mouse familiar with the experimenter.

### Chemicals

RU24969 (HY-16688; MedChemExpress, Monmouth Junction, USA), 8-OH-DPAT (B6337, Houston, USA), and MCPP (125180; Sigma-Aldrich, Saint Louis, USA) were dissolved in 0.9% saline. RU24969 and MCPP were injected intraperitoneally, whereas 8-OH-DPAT was subcutaneously administered. Drug doses were selected based on previous dose–response studies [11–15]. Drugs were injected at a volume of 20 ml/kg and 5 ml/kg for intraperitoneal and subcutaneous administration, respectively.

### Experimental procedures

#### Experiment 1: behavioral experiments

After adapting to the experimental environment for 30 min, different groups of mice received acute injections of RU24969 (10 mg/kg), 8-OH-DPAT (3 mg/kg), and MCPP (0.1 mg/kg), and their respective control groups were injected with the same volume of saline. Different drugs have very different onset of action [12–14, 16, 17]. Based on these literatures, 5 min (for RU24969 and 8-OH-DPAT) or 20 min (for MCPP) after injection, mice were tested in the circling behavior test, self-grooming test, induced-grooming test, open-field test (OFT), marble-burying test (MBT), and novel object recognition test (NOR) on separate days, different behavioral tests were conducted at least 2 days apart. Food and water were not present, and the luminance intensity was maintained at 5w during all the behavioral procedures.

##### Circling behavior test

An open field was used to evaluate the circling behavior. The apparatus consisted of a non-porous plastic box with side lengths of 35 cm and a height of 25 cm. The circling behavior was assessed as described in a previous study [14]. The animals were placed in the center of the open field, the bouts and durations of circling were determined for 20 min using TopScan Version 3.0 (Clever Sys, Inc., Reston, USA) and SuperMaze (XR-Xmaze, Softmaze, Shanghai, Chian). *Circling* calculation program in TopScan was used to quantify circling behavior. The mouse rotation angle equal to 360° within the setting range of movement speed was recorded as one circling.

##### Self-grooming test

The protocol of the self-grooming test was adapted from [18]. The mice were placed in a square transparent mouse cage without food, water, and litter. After acclimation for 10 min, SuperMaze was used for video recording and TopScan for analyzing their grooming behavior within 20 min.

##### Induced-grooming test

The mouse was placed in a cage as described for the self-grooming test. After 10 min, the mouse was gently sprayed with a watering can, and the subsequent grooming behavior of the mouse was recorded within 20 min for further analyses [19]. The grooming of any part of the body was constituted a grooming event in Self-grooming and Induced-grooming test.

##### Open-field test

An open field was used to evaluate anxiety behavior [20]. Animals were placed in the center of the open field as described for the circling behavior test, and the total time in the inner zone and the total distance covered were determined over 10 min.

##### Marble-burying test

The MBT was carried out in a square box (31 cm × 23 cm × 16 cm), and a layer of 5 cm corncob litter was laid on the bottom of this box, flattened, and slightly compacted. Afterward, 20 black marbles were evenly distributed on the surface of the corncob litter. During the experiment, mice were gently placed into the box and were quickly removed after 30 min. The number of buried marbles (more than two-thirds of the volume was buried in the corncob) was calculated [21].

##### Novel object recognition test

The NOR experiment was performed in the open-field device described above. The NOR experiment was carried out over 3 consecutive days [22, 23]. For habituation on the first day, mice were placed in the open field without any objects for 5 min. For the familiarization session after 24 h, two objects with the same shape and color were placed into the box, both 5 cm away from the wall. The mouse was placed into the box and removed after becoming familiar with these objects for 10 min. After an additional 24 h, two objects were placed in the box for the test session. One was the old object used in the previous familiarization session, whereas the other was a novel object with a different shape, color, and texture. The mouse behavior was recorded for 5 min. The interaction between the animal and the objects was measured according to the time the mouse spent sniffing the novel object within a range of 2 cm. The discrimination index calculated as the interaction time with the new object divided by the sum of the interaction times for the two objects was used to measure the interaction between the mouse and the novel object.

#### Experiment 2: immunofluorescence

Immunofluorescence staining was performed on different days with behavior tests. The procedures were described in previous work [24]. Briefly, 2 h after the acute drug injection, the mice were anesthetized with tribromoethanol (20 mg/kg), perfused with phosphate-buffered saline (PBS), and 4% paraformaldehyde (AR1068; Boster Biological Technology, Wuhan, China) for pre-fixation. The brain tissue was removed, placed in 4% paraformaldehyde, and stored at 4 °C for 24 h, then dehydrated with 20% and 30% sucrose solutions for 2 days. The OTC-embedded tissue was cut into 40-μm sections using a freezing microtome (CM1950; Leica, Wetzlar, Germany). After permeabilization and blocking, the sections were incubated with primary anti-c-fos antibody (1:500, rabbit, #2250; Cell Signaling Technology, Danvers, USA) at 4 °C for 20 h and then washed three times with PBS. Afterward, the sections were incubated with the secondary Alexa Fluor488-conjugated donkey anti-rabbit antibody (1:500, A21208; Invitrogen, Carlsbad, USA) at room temperature for about 2 h and washed with PBS. To stain the nuclei, 4′,6′-diamidino-2-phenylindole (DAPI, 0.1 μl/ml) was used for about 5 min. Sections were washed with PBS and covered on a glass slide. Brain slices were imaged using a confocal microscope (LSM 880 with Airyscan; Zeiss, Jena, Germany).

#### Statistical analysis

Prism software (GraphPad 8.0) was used for the statistical analysis. c-fos expression was analyzed using the ImageJ software (National Institutes of Health, Bethesda, MD, USA). The student’s *t*-test was used to compare the statistical difference between two groups in behavioral experiments and immunofluorescence. In the NOR, the exploration time of the novel and the old object in the same group was compared using the paired *t*-test. Data are expressed as the mean ± SEM. Significant differences were defined as p < 0.05.

## Results

### Ru24969-treated mice showed repetitive circling, memory impairment, and anxiety

In previous studies, pharmacological OCD models were mainly derived from rats [10, 11]. We established OCD mouse models by administration of RU24969, 8-OH-DPAT, and MCPP to complement these rat models.

**Fig. 1:**
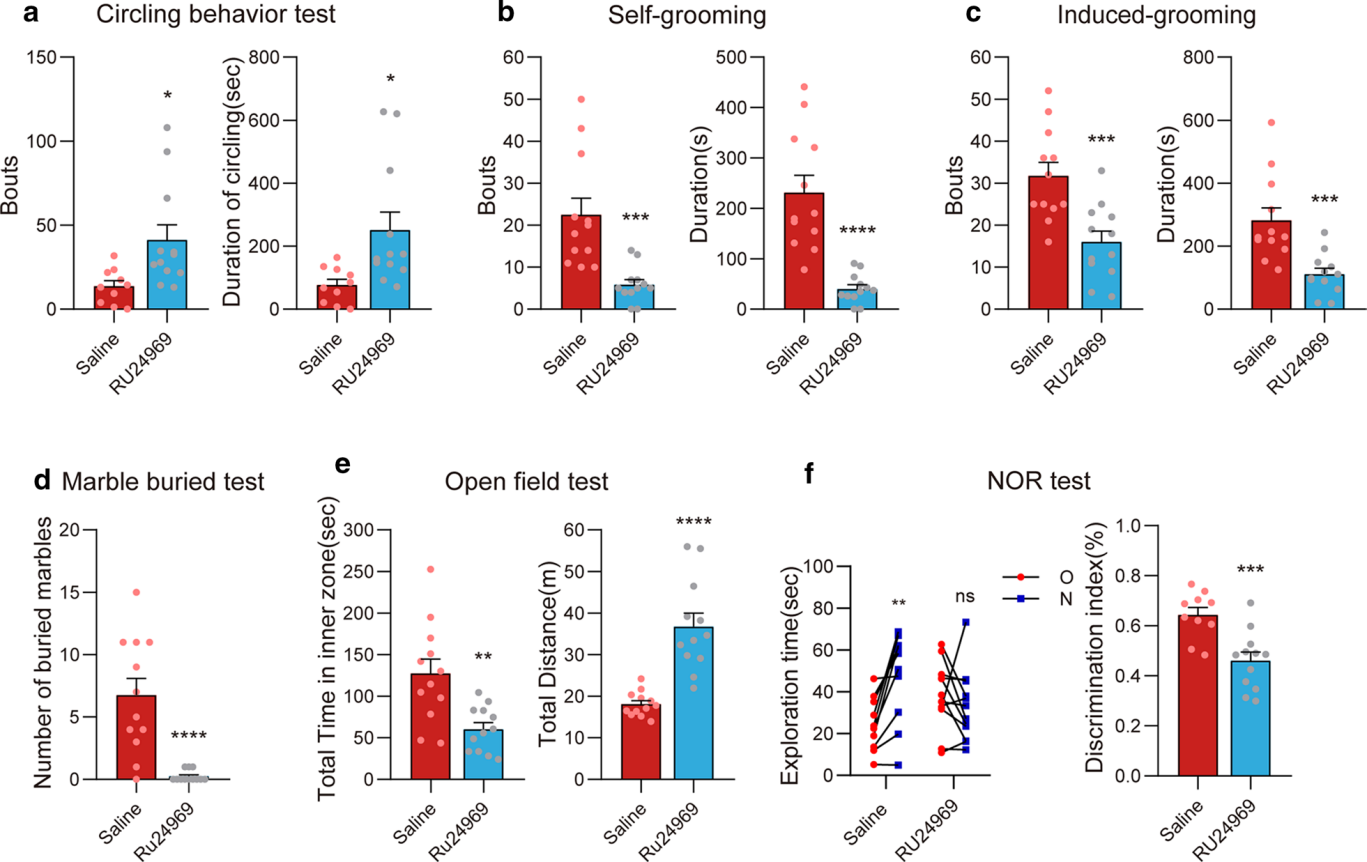
Ru24969-treated mice showed repetitive circling behavior, memory impairments, and anxiety. Comparison between RU24969- and saline-treated mice regarding bouts and duration in the circling behavior (**a**), self-grooming (**b**), and spray-induced grooming (**c**) tests; the number of buried marbles in the MBT (**d**); bouts and duration in the OFT (**e**); and exploration time and discrimination index in the NOR (**f**). Data are expressed as the mean ± SEM. Unpaired t-test, except for the exploration time in the NOR using the paired *t*-test: **p < 0.01, ***p < 0.001, ****p < 0.0001

The effects of RU24969 treatment on mouse behavior were summarized in Fig. 1. We examined circling, self-grooming, spray-induced grooming, and MBT in RU24969-treated mice. Acute treatment with RU24969 increased both bouts (*t*_(20)_ = 2.68, *p* < 0.05) and duration (*t*_(20)_ = 2.69, *p* < 0.05) of circling, compared with saline-treated mice (Fig. 1a). RU24969-treated mice showed repeated circling around the edges of the open field, whereas saline-treated mice moved in random directions. These results suggest that RU24969 mice exhibited repetitive, stereotyped behavior. Probably due to the long-term repeated movement in circles in the cage, and thus ignoring the marbles and reducing the grooming, RU24969-treated mice did not show increased OCD-like behavior in MBT and grooming tests. Instead, the numbers of buried marbles and grooming bouts were decreased (Fig. 1b–d) (self-grooming, bouts: *t*_(22)_ = 4.08, *p* < 0.001; self-grooming, duration: *t*_(22)_ = 5.44, *p* < 0.0001; induced-grooming, bouts: *t*_(22)_ = 3.84, *p* < 0.001; induced-grooming, duration: *t*_(22)_ = 3.89, *p* < 0.001; MBT: *t*_(22)_ = 4.82, *p* < 0.0001; Fig. 1b–d).

The OFT is frequently used to measure anxiety and locomotion levels in rodents [20]. In the OFT, the total time in the inner zoom was reduced in the RU24969-treated group (*t*_(22)_ = 3.49, *p* < 0.01), and the total distance was increased (*t*_(22)_ = 5.69, *p* < 0.0001; Fig. 1e), indicating that RU24969-treated mice exhibited anxiety and hyperlocomotion. In the NOR test, the control group mice showed a preference for novel objects in the test session (*t*_(9)_ = 4.35, *p* < 0.01), whereas the RU24969-treated mice did not show this preference (*t*_(11)_ = 0.99, *p* = 0.34; Fig. 1f). The discrimination index was significantly lower in the RU24969 group (*t*_(20)_ = 4.01, *p* < 0.001; Fig. 1f). These results suggest that RU24969 administration led to the impairment of recognition memory.

### MCPP induced repetitive self-grooming in mice

**Fig. 2:**
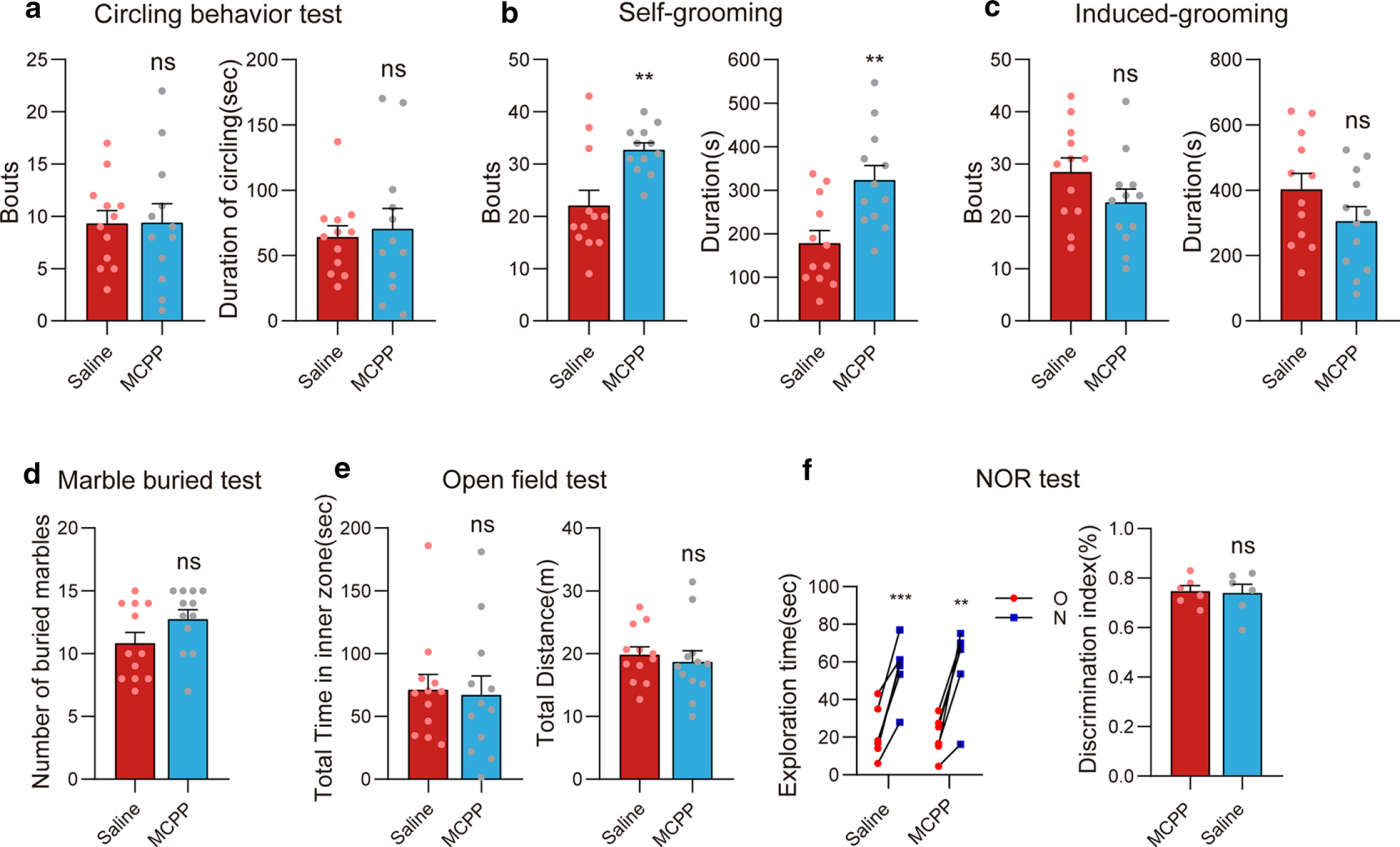
MCPP induced repetitive self-grooming behavior but no other behavioral abnormalities in mice. Bouts and duration in the circling behavior (**a**), self-grooming (**b**), and induced-grooming (**c**) tests; the number of buried marbles in the MBT (**d**), bouts and duration in the OFT (**e**), and exploration time and discrimination index in the NOR (**f**) comparing MCPP- with saline-treated control mice. Data are expressed as the mean ± SEM. Unpaired *t*-test, except for the exploration time in the NOR using the paired *t*-test: *p < 0.05, **p < 0.01, ***p < 0.001, ****p < 0.0001

Previous studies reported that MCPP induced repetitive self-grooming in rats [16, 18]. Could the same or distinct behavioral phenotypes be induced in mice? To answer this question, we performed behavioral tests in MCPP-treated mice. Our results showed that, similar to the rats, MCPP-treated mice exhibited increased self-grooming (Fig. 2a–f). Both grooming bouts (*t*_(22)_ = 3.33, *p* < 0.01) and duration (*t*_(22)_ = 3.31, *p* < 0.01; Fig. 2b) were increased, suggesting that MCPP could induce over-grooming in mice. However, MCPP-treated mice did not show a difference in other behavioral tests than saline-treated mice (Fig. 2a, c–f).

### 8-OH-DPAT-treated mice exhibited excessive grooming and memory impairment but not anxiety

**Fig. 3:**
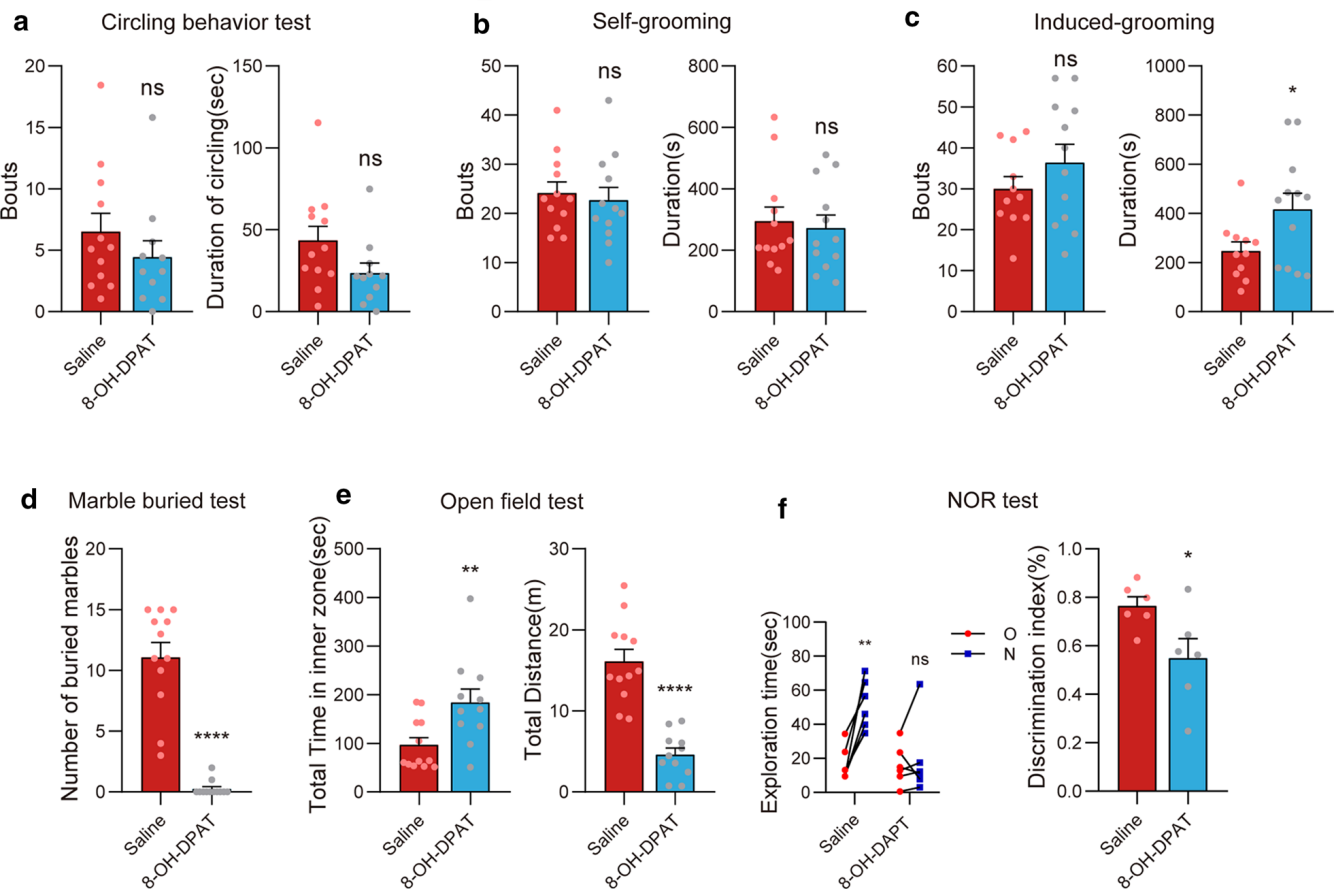
Excessive grooming and memory impairment, but no anxiety, in 8-OH-DPAT-treated mice. Bouts and duration in the circling behavior (**a**), self-grooming (**b**), and induced-grooming (**c**) tests; the number of buried marbles in the MBT (**d**), bouts and duration in the OFT (**e**), and exploration time and discrimination index in the NOR (**f**) comparing 8-OH-DPAT mice with saline-treated mice. Data are expressed as the mean ± SEM. Unpaired t-test, except for the exploration time in the NOR using the paired *t*-test: *p < 0.05, **p < 0.01, ****p < 0.0001

8-OH-DPAT is a commonly used reagent to induce OCD-like behavior in the rat [10]. When we applied it to mice (Fig. 3a–f), we found that the duration of spray-induced grooming was significantly increased (*t*_(23)_ = 2.22, *p* < 0.05), although the number of bouts was not different compared with the saline-treated group (*t*_(23)_ = 1.19, *p* = 0.25; Fig. 3c). Surprisingly, in self-grooming test, 8-OH-DPAT-treated mice did not exhibit over-grooming (bouts: *t*_(22)_ = 0.44, *p* = 0.67; duration: *t*_(22)_ = 0.36, *p* = 0.72; Fig. 3b). Indeed, self-grooming and spray-induced grooming represent very different grooming forms; anomalies in one form were not necessarily accompanied by deficits in the other [25]. We found no significant alteration in circling, either (bouts: *t*_(21)_ = 1.04, *p* = 0.31; duration: *t*_(21)_ = 1.85, *p* = 0.08; Fig. 3a).

Notably, the number of buried marbles was decreased in the 8-OH-DPAT-treated group (*t*_(22)_ = 8.86, *p* < 0.0001; Fig. 3d). Besides, 8-OH-DPAT also caused reduced locomotion as shown in the OFT; the total distance was lower in the 8-OH-DPAT group than in the control group (*t*_(21)_ = 6.67, *p* < 0.0001), whereas the total time in the inner zone was increased (*t*_(21)_ = 2.86, *p* < 0.01; Fig. 3e). This suggests the motion inhibition and lack of anxiety in 8-OH-DPAT-treated mice. Noteworthy, 8-OH-DPAT-treated mice also presented memory impairments in the NOR; the discrimination index of 8-OH-DPAT-treated mice was significantly lower than that of the saline-treated group (*t*_(10)_ = 2.42, *p* < 0.05; Fig. 3f).

### Increased c-fos expression in the OFC, ACC, PrL, IL, CPu, NAc, hypothalamus, BSTLD, and IPACL in RU24969-treated mice

**Fig. 4:**
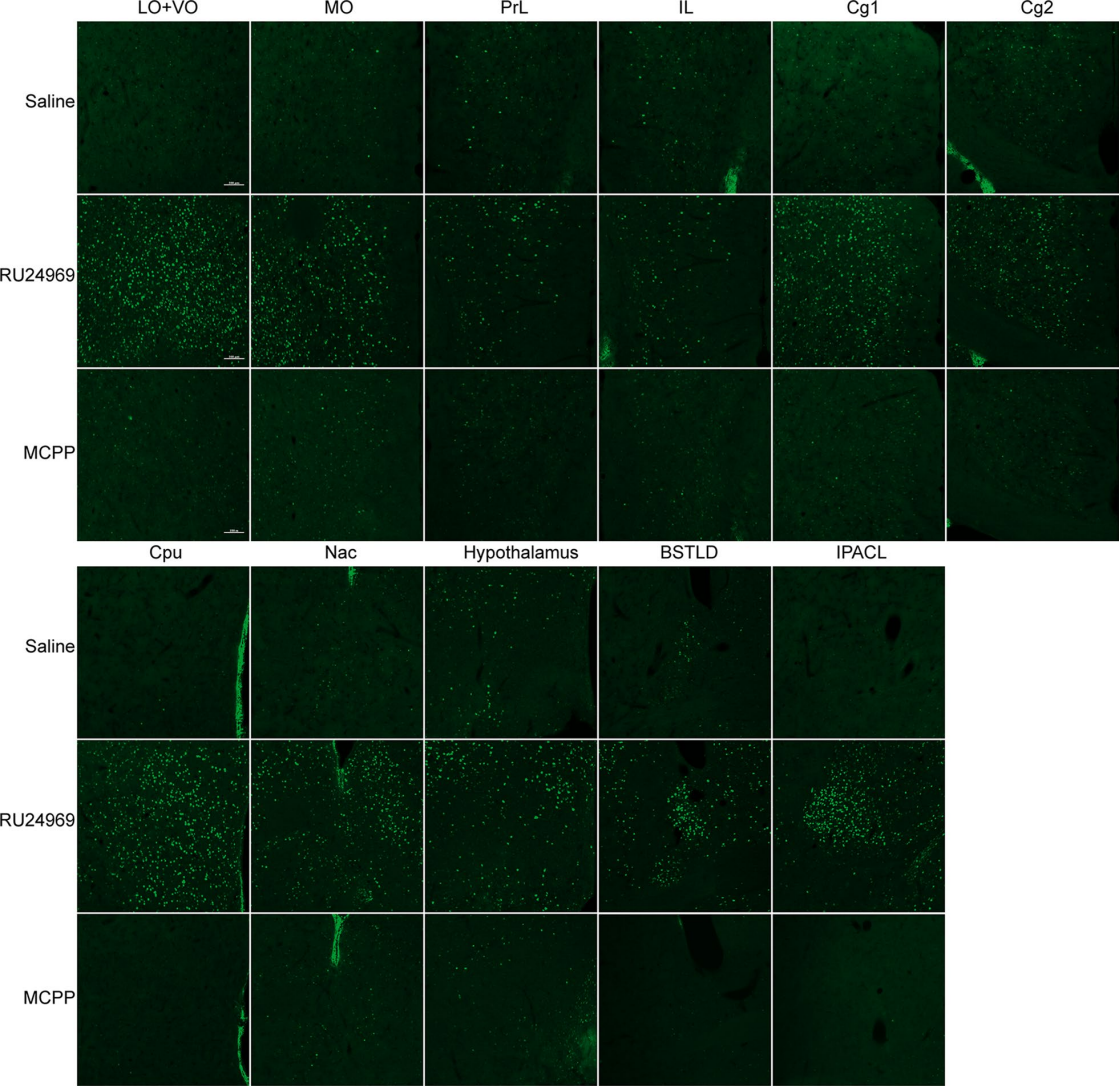
Mapping c-fos expression in RU24969- and MCPP-treated mice. Representative immunofluorescence micrographs depicting c-fos expression in the OFC, PrL, IL, ACC, CPu, NAc, hypothalamus, BSTLD, and IPACL in RU24969-, MCPP- and saline-treated mice (Scale bars: 100 μm)

The distinct behavioral traits observed above suggest that each model might involve the activation of different brain regions. To validate this hypothesis, we used c-fos expression to indicate the neuronal activation in different brain regions. We found that RU24969 induced higher c-fos expression in many brain areas, including OFC, ACC, PrL, IL, CPu, nucleus NAc, hypothalamus, BSTLD, and IPACL (Fig. 4a, b, Additional file 1: Fig. S1). Noteworthy, dysfunctions of the OFC, including the lateral orbital cortex (LO), ventral orbital cortex (VO), and medial orbital cortex (MO), and ACC, have been implicated in the etiology of OCD in previous studies [11]. Consistently, all of these regions showed increased c-fos expression in our RU24969-treated mice (LO + VO: *t*_(16)_ = 4.30, *p* < 0.001; MO: *t*_(16)_ = 2.78, *p* < 0.05; Cg1: *t*_(16)_ = 3.42, *p* < 0.01; Cg2: *t*_(16)_ = 3.11, *p* < 0.01). The OFC and ACC are involved in many important neural functions, such as decision making, planning, inhibition of responses, and error monitoring. Indeed, in OCD patients, almost all of these functions are impaired [5, 11]. The PrL and IL, components of the medial prefrontal cortex (mPFC), showed increased c-fos expression in the RU24969-treated group (PrL: *t*_(16)_ = 4.15, *p* < 0.001; IL: *t*_(16)_ = 2.30, *p* < 0.05), which may suggest the dysfunction in recognition memory, attention, and decision making [26]. In the CPu, c-fos was also highly expressed in RU24969-treated mice, whereas hardly detectable in saline-treated mice (*t*_(16)_ = 6.92, *p* < 0.0001). This was consistent with the findings that CPu’s functions in learning, memory, action selection, and goal-directed actions were impaired in OCD patients [5]. The accumbens nucleus, shell (AcbSh) and accumbens nucleus, core (AcbC) are two regions of the NAc and participate in the regulation of reinforcement learning [27]. They also exhibited higher c-fos expression in RU24969-treated mice (AcbSh: *t*_(16)_ = 5.16, *p* < 0.0001; AcbC: *t*_(16)_ = 3.94, *p* < 0.01). The BSTLD, a brain region related to the regulation of anxiety and acute stress [28], showed increased c-fos expression in RU24969-treated mice (*t*_(16)_ = 5.73, *p* < 0.0001). Furthermore, we found that c-fos was elevated in the IPACL in RU24969-treated mice (IPACL: *t*_(16)_ = 10.63, *p* < 0.0001; IL: *t*_(16)_ = 3.94, *p* < 0.01). The IPACL receives projections from the amygdala [29], but its functions related to OCD have not been clarified. Both control and RU24969-treated mice showed robust c-fos expression in the hypothalamus, a brain region that responds to multiple stimulations including stress and fear [30, 31], but the c-fos level was significantly higher in the RU24969-treated group (*t*_(16)_ = 6.93, *p* < 0.0001).

### Neuronal activity is not significantly increased in MCPP-treated mice

**Fig. 5:**
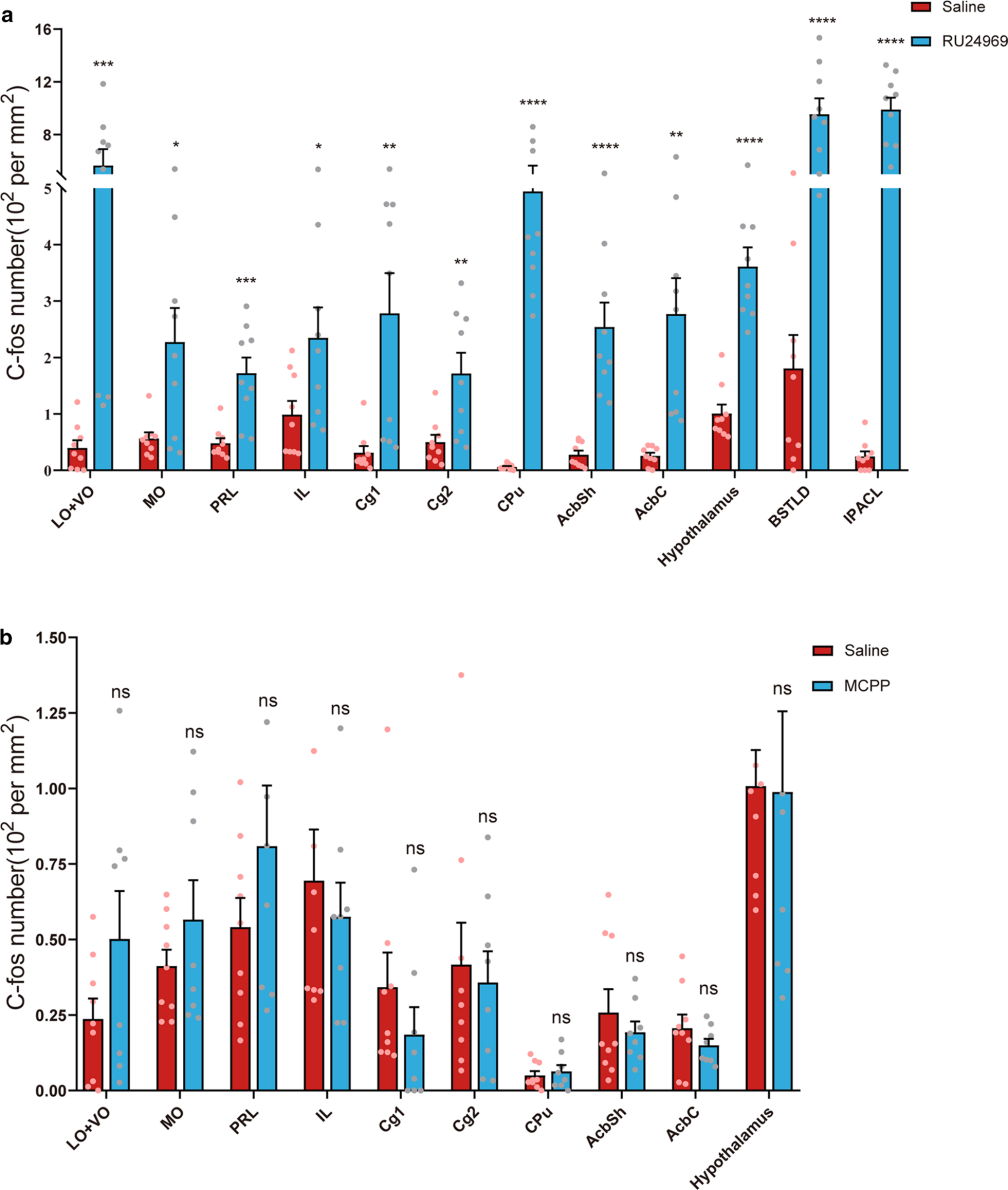
Quantification of the c-fos expression in RU24969- and MCPP-treated mice. **a** In RU24969-treated mice, c-fos expression levels were increased in the OFC, PrL, IL, CPu, NAc, hypothalamus, BSTLD, and IPACL. **b** There were no significant differences between MCPP- and saline-treated mice regarding the c-fos expression in the OFC, PrL, IL, CPu, NAc, hypothalamus, BSTLD, and IPACL. Data are expressed as the mean ± SEM. Unpaired *t*-test: *p < 0.05, **p < 0.01, ***p < 0.001, ****p < 0.0001

Next, we investigated MCPP-induced c-foc expression in mice brains. Surprisingly, after MCPP injection, we found that limited brain areas expressed c-fos with a weak signal. When we compared it with the saline group, there were no statistically significant differences (Fig. 5b). No detectable changes were seen in the cortical brain areas that we examined.

### Increased c-fos expression in the ACC, PrL, IL, OFC, AcbSh, and hypothalamus in 8-OH-DPAT-treated mice

**Fig. 6:**
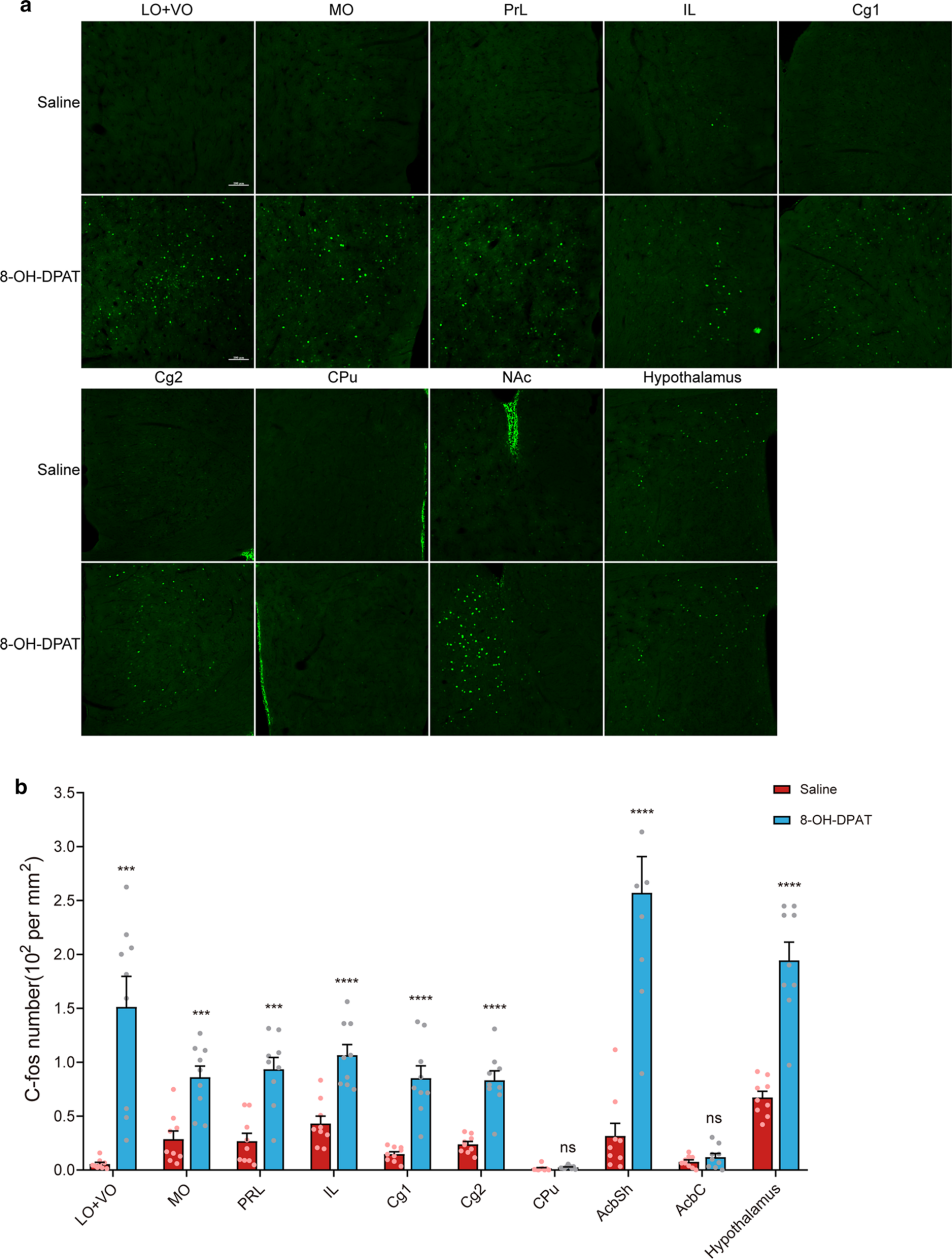
The c-fos expression in the brain of 8-OH-DPAT-treated mice. **a** Representative immunofluorescence micrographs depicting the c-fos expression levels in the ACC, PrL, IL, OFC, AcbSh, and hypothalamus. Expression levels were increased in 8-OH-DPAT-treated mice compared with saline-treated control mice, whereas c-fos expression levels in the Acbc and CPu were not significantly different (Scale bars: 100 μm). **b** Quantification of the c-fos expression in the OFC, PrL, IL, ACC, CPu, NAc, and hypothalamus in 8-OH-DPAT and saline-treated mice. Data are expressed as the mean ± SEM. Unpaired *t*-test: ***p < 0.001, ****p < 0.0001

The brain regions expressing c-fos after 8-OH-DPAT administration were similar but not identical to those after RU24969 administration. In 8-OH-DPAT-treated mice, higher c-fos expression were detected in the ACC, PrL, IL, OFC, AcbSh, and hypothalamus (Cg1: *t*_(16)_ = 5.99, *p* < 0.0001; Cg2: *t*_(16)_ = 6.50, *p* < 0.0001; PrL: *t*_(16)_ = 5.02, *p* < 0.0001; IL: *t*_(16)_ = 5.25, *p* < 0.0001; LO + VO: *t*_(16)_ = 5.13, *p* < 0.0001; MO: *t*_(16)_ = 4.50, *p* < 0.001; AcbSh: *t*_(16)_ = 6.32, *p* < 0.0001; hypothalamus: *t*_(16)_ = 7.15, *p* < 0.0001; Fig. 6a, b). These results indicate that the excessive spay-induced grooming observed in 8-OH-DPAT-treated mice might be mediated by the activation of these brain areas.

### OCD mouse models exhibited a distinct pattern of the activated brain regions

**Fig. 7:**
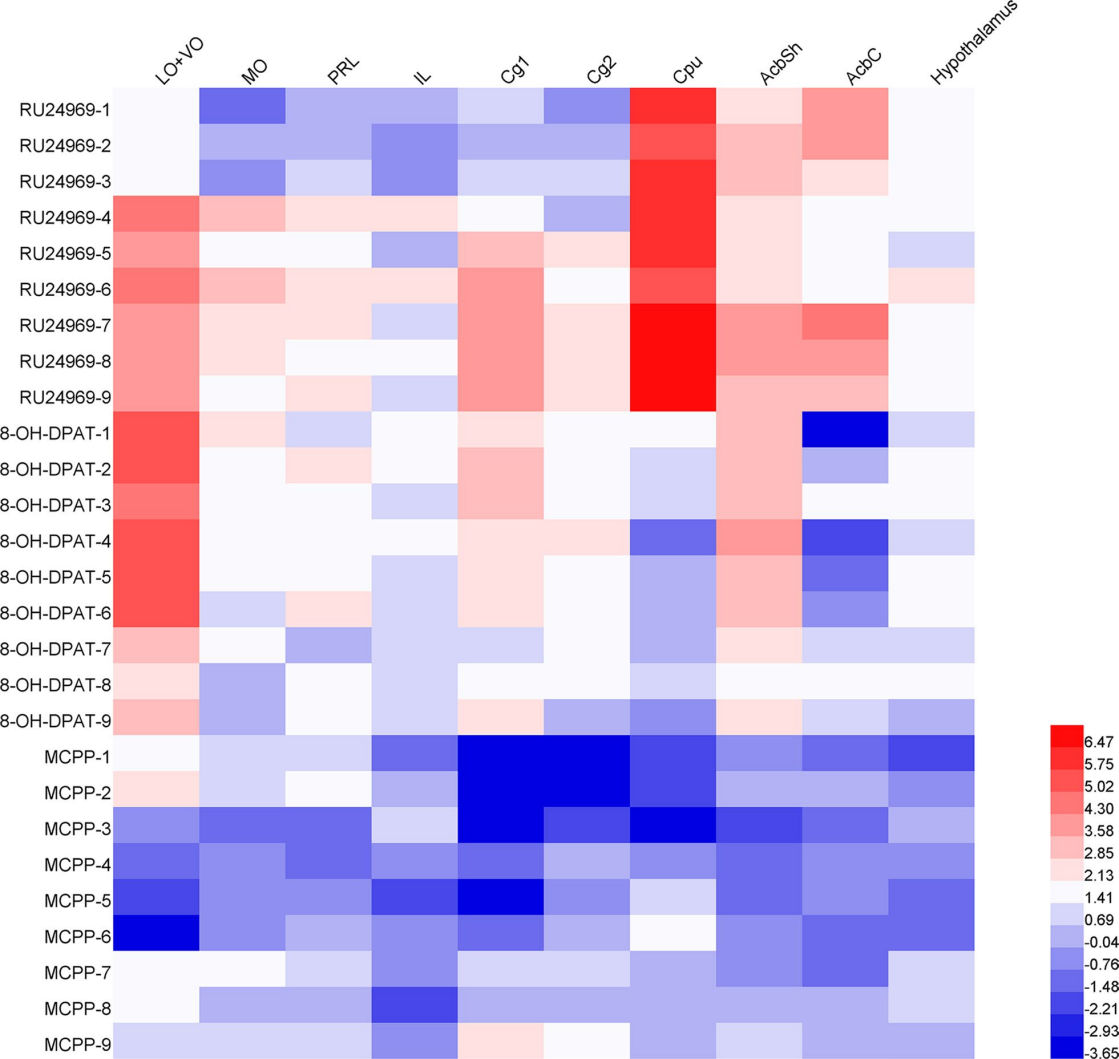
Heatmap of the c-fos expression in the OCD models. The color range represents a logarithm of 2 for the ratio of each mouse to the corresponding control average $\left( {\log_{2} \frac{{{\text{each }}\,{\text{mouse}}\,{\text{of}}\,{\text{OCD models}}}}{{{\text{control }}\,{\text{average}}}}} \right)$. Red indicates high expression

To more intuitively compare the changes in the activation of brain regions in the three OCD mouse models, we constructed a heatmap of c-fos expression (Fig. 7). The heatmap clearly illustrated the distinct pattern of the activated brain regions, in which increased c-fos were detected in the OFC, ACC, PrL, IL, CPu, NAc, and hypothalamus in RU24969-treated mice, and the OFC, ACC, PrL, IL, AcbSh, and hypothalamus in 8-OH-DPAT-treated mice. In contrast, no significant increase was detected in our interested brain regions in MCPP-treated mice.

## Discussion

Pharmacological animal models of OCD have been widely used in previous studies [10, 11]. However, most of the published studies used rats instead of mice. In this report, three mouse models of OCD were established to provide more options for drug-induced OCD models and offered more possibilities for researchers to explore the mechanisms of OCD.

RU24969, 8-OH-DPAT, and MCPP all belong to serotonin receptor agonists. The choice of these reagents was made based on previous clinical experience with OCD medications and hypothesized involvement of the serotonin system in OCD. They all augment the sensitivity of 5-HT receptor subtypes, which caused OCD [13].

The dysfunction of 5-HT receptor 1B (5-HT1B) in OCD has been suggested in previous studies. For example, in pharmacological studies, the use of 5-HT1B agonists aggravated symptoms in patients with OCD, and mutations of the serotonin transporter *SCL6A4* were associated with OCD [14]. As serotonin 5-HT1A/1B receptor agonist, RU24969 treatment in rats induced locomotor stereotypy, prepulse inhibition (PPI) deficits, and impairments in delayed alternation, all related to common signs and symptoms in OCD patients [12]. Consistently, in the present study, RU24969 administration induced repetitive circling behavior in mice (Additional file 2: Fig. S2). Moreover, the mice exhibited anxiety in the OFT. Impaired memory is one of the cognitive dysfunctions of OCD [32]. In the NOR, RU24969-treated mice had a decreased time to explore novel objects indicative of memory impairment, further confirming the validity of this model.

8-OH-DPAT, a 5-HT receptor 1A (5-HT1A) agonist, could bind to its receptor with high selectivity [33]. 8-OH-DPAT-treated animals were commonly used as an OCD model. In the spontaneous alternation behavior test, the animals repeatedly selected the same arm, similar to the OCD perseveration symptoms [34]. Importantly, treatment with selective serotonin reuptake inhibitors could eliminate this repetitive behavior in the 8-OH-DPAT rat model [35]. However, other OCD-like behavioral tests have been rarely tested in the rat model. In the present study, 8-OH-DPAT administration induced spray-induced grooming in mice. Interestingly, self-grooming behaviors were not increased in 8-OH-DPAT mice compared to control. This may be due to the necessity of a trigger to induce OCD-like behavior in this model, similar to patients with excessive washing or cleaning behavior whose symptoms were triggered by contamination stimuli in clinical [4]. Thus, the OCD-like behaviors in 8-OH-DPAT-treated mice differed from those in RU24969-treated mice. On the other side, similar to RU24969-treated mice, 8-OH-DPAT-treated mice showed recognition memory impairments in the NOR, whereas anxiety was not detected in the OFT, which might be related to a lack of related stimuli in these experiments.

Administration of MCPP, which binds to 5-HT1A and 5-HT2C receptors, could aggravate symptoms in patients with OCD [13, 36]. In a preclinical study, the administration of MCPP blocked the beneficial effects of fluoxetine for OCD treatment. Moreover, MCPP could induce the occurrence of repetitive stereotypes such as increasing the number of buried marbles in the MBT [13], promoting directional persistence in spontaneous alternation behavior [11], and reinforcing self-grooming [16]. In the current study, MCPP-induced OCD-like behaviors comprised excessive self-grooming behavior, whereas the induced-grooming test showed no significant differences to control mice. These results contrasted with those in 8-OH-DPAT mice. Self-grooming and spray-induced grooming are different grooming forms [25]. Self-grooming is spontaneous; thus, excessive self-grooming in MCPP mice resembles trichotillomania (hair-pulling disorder) appearing spontaneously or under high pressure. Besides, the MCPP model presented no anxiety or memory impairment according to the OFT and NOR. Most patients with OCD chose to obey obsessive–compulsive thoughts and perform obsessive–compulsive behaviors to alleviate the anxiety caused by obsessive–compulsive thoughts and impulses. Thus, the reduction of anxiety in MCPP-treated mice may be due to the performance of obsessive–compulsive behavior.

Together, the three newly established OCD mouse models exhibited distinct OCD-like behavioral traits and presented different levels of anxiety and memory dysfunction.

The expression of c-fos is one of the commonly used indicators to measure the activity of neurons because repetitive action potentials were often accompanied by neuronal c-fos expression [37]]. Based on the drugs’ pharmacological properties and the expression of the receptors, we speculate that the increase in c-fos expression was majorly raised from neurons. All three drugs are 5-HT receptor agonists and can act on almost all neuronal cells. The distribution of their corresponding receptors is wide. 5-HT 1A receptors were expressed in the pyramidal neurons of the cortex, hippocampus, and raphe nuclei and the cholinergic neurons in the septum [38–40]. 5-HT 1B receptors were also expressed in the medium spiny neurons in the caudate putamen, probably GABAergic [41, 42]. 5-HT 2C receptors were expressed in most GABAergic cells. Astrocyte was also reported to express 5-HT receptors; thus, the astrocyte could also be a source for the c-fos signal. Previous studies proposed that the dysfunction of CSTC loops is related to different cognitive-affective processes in OCD. The brain regions chosen in this study belong to the CSTC loop (except for BSTLD and IPACL). Here, neuronal activity was increased in the OFC, PrL, IL, ACC, CPu, NAc, BSTLD, IPACL, and hypothalamus of RU24969-treated mice. In 8-OH-DPAT-treated mice, activated brain regions included the ACC, PrL, IL, OFC, and AcbSh (Additional file 3: Table S1). This was similar to previous studies in mouse and rats [33, 43–45] that 5-HT1B receptors are distributed in the striatum, cerebellum, and basal ganglia, 5-HT1A receptors were distributed in the neocortex, olfactory areas, hippocampal formation, cortical subplate, pallidum, hypothalamus, and mesencephalic raphe nuclei, although the brain regions with increased neuronal activity in these two model mice did not completely coincide with those expressing 5-HT1A or 5-HT1B receptors. One possible reason is that the OCD-like behaviors were not induced by directly activating all relevant brain regions containing 5-HT1A or 5-HT1B receptors, but acting within the same receptor-associated brain region instead, therefore indirectly increasing the neuronal activity of downstream brain regions and causing the occurrence of OCD-like behaviors. Notably, most of the activated brain regions were related to the CSTC loop elements corresponding to the postulated CSTC loop dysfunction in OCD.

In mice and rats, the 5-HT receptor 2C (5-HT2C) is mainly distributed in the choroid plexus and other areas, including the nucleus accumbens, patches of the caudate-putamen, the olfactory tubercle, claustrum, septum, cingulate cortex, amygdala, dentate gyrus, periaqueductal gray, and entorhinal cortex [37, 44, 46]. Interestingly, after injection with the 5-HT2C agonist MCPP, we observed a weak c-fos expression in the mouse brains, with no difference to the control group. Even after increasing the dose, we obtained the same results (data not shown). In this MCPP model, mice only showed excessive self-grooming behavior, whereas all other behaviors appeared normal. To a certain extent, this was consistent between behavioral and c-fos expression. We speculate that MCPP injection might cause inhibition in some brain regions, which could not be reflected by c-fos elevation. Indeed, previous studies found that OCD patients have decreased activity in certain brain regions [47, 48].

## Conclusion

Here, we established three different OCD mouse models with distinct OCD-like behaviors, accompanying dysfunctions in different brain regions. Compared to controls, RU24969- and 8-OH-DPAT-treated mice demonstrated enhanced activation in brain regions mostly belonging to the CSTC circuit, although some of the activated brain regions differed between the two models. Neuronal activity was not significantly increased in MCPP-treated mice. These results implied that targeted and individualized treatment should be performed in OCD patients with distinct symptoms, and interventional treatment targeting the corresponding specific brain regions may improve the therapeutic effects.

## Supplementary Information

**Additional file 1: Figure S1.** Coronal section region of c-fos expression (green) of in RU24969-, MCPP-treated mice (**A**), and 8-OH-DPAT-treated mice (**B**).

**Additional file 2: Figure S2.** Heat map of OFT test. (**A**) RU24969-treated mice showed repeated circling around the edges of the open field. (**B**) MCPP-treated mice moved in random directions similar to the saline group. **(C)** 8-OH-DPAT-treated mice reduced locomotion while spent more time in the inner zone.

**Additional file 3: Table S1.** cFos expression of the models (mean ± SEM).

## Abbreviations

5-HT: 5-Hydroxytryptamine
8-OH-DPAT: 8-Hydroxy-DPAT hydrobromide
AcbC: Accumbens nucleus, core
AcbSh: Accumbens nucleus, shell
ACC: Anterior cingulate cortex
BSTLD: Bed nucleus of the stria terminalis, lateral division, intermediate part
Cg1/2: Cingulate cortex, area 1/2
CPu: Caudate-putamen
CSTC: Cortico–striato-thalamo-cortical
IL: Infralimbic cortex
IPACL: Interstitial nucleus of the posterior limb of the anterior commissure, lateral part
LO: Lateral orbital cortex
MBT: Marble-burying test
MCPP: 1-(3-Chlorophenyl) piperazine hydrochloride-99%
MO: Medial orbital cortex
mPFC: Medial prefrontal cortex
NAc: Nucleus accumbens
NOR: Novel object recognition test
OCD: Obsessive–compulsive disorder
OFC: Orbitofrontal cortex
OFT: Open-field test
PBS: Phosphate-buffered saline
PPI: Prepulse inhibition
PrL: Prelimbic cortex
VO: Ventral orbital cortex
