# Supplementary figures and images for: Distinct behavioral traits and associated brain regions in mouse models for obsessive–compulsive disorder
Source: Behav Brain Funct. 2021 May 18;17:4. doi: 10.1186/s12993-021-00177-x (PMC8132448; doi:10.1186/s12993-021-00177-x)

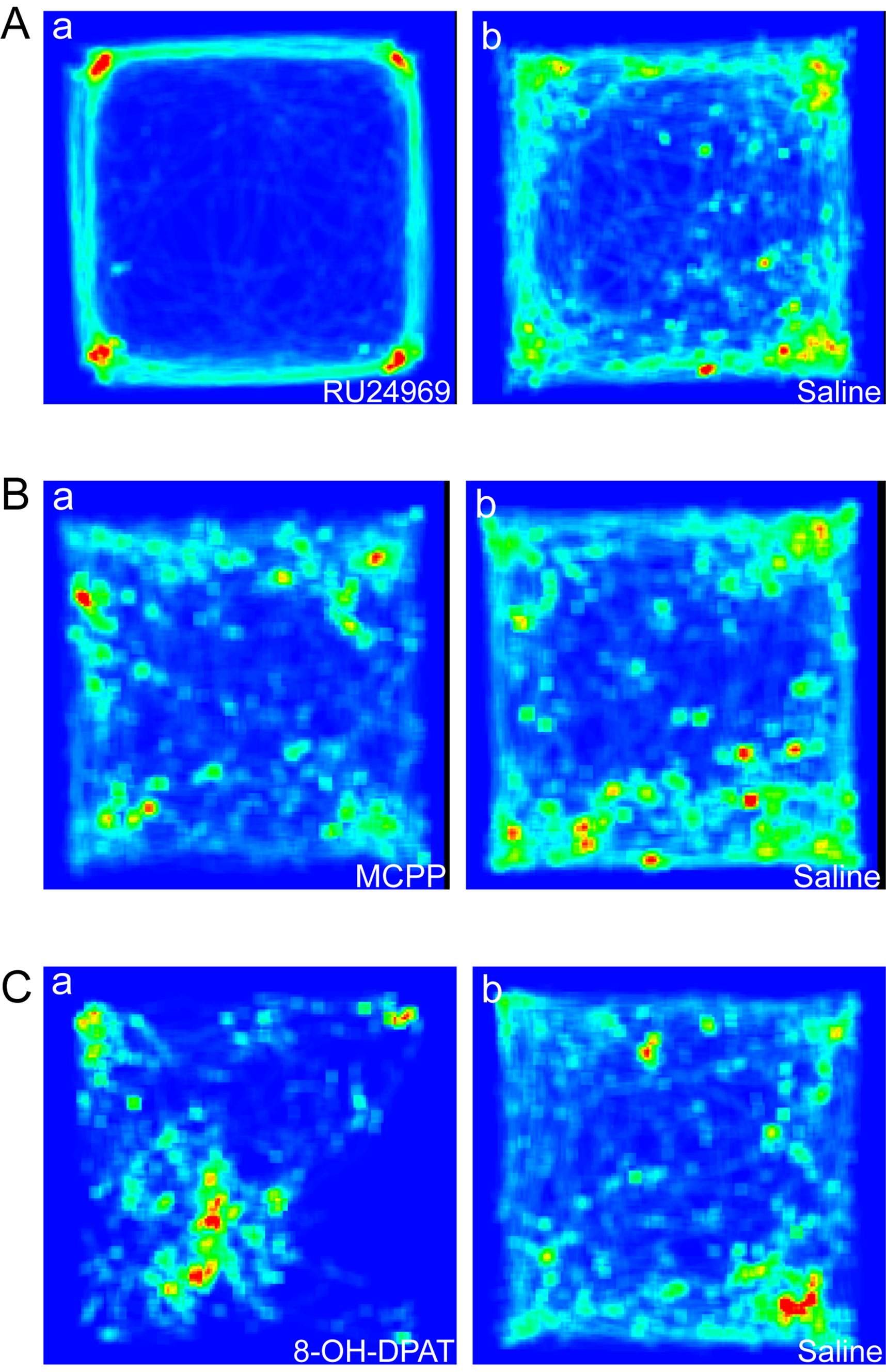

Supplement: Supplementary file 2 — Additional file 2: Figure S2. Heat map of OFT test. (A) RU24969-treated mice showed repeated circling around the edges of the open field. (B) MCPP-treated mice moved in random directions similar to the saline group. (C) 8-OH-DPAT-treated mice reduced locomotion while spent more time in the inner zone. [file 12993_2021_177_MOESM2_ESM.tif]
