# Supplementary material for: Distinct behavioral traits and associated brain regions in mouse models for obsessive–compulsive disorder
Source: Behav Brain Funct. 2021 May 18;17:4. doi: 10.1186/s12993-021-00177-x (PMC8132448; doi:10.1186/s12993-021-00177-x)
Supplement: Supplementary file 3 — Additional file 3: Table S1. cFos expression of the models (mean ± SEM). [file 12993_2021_177_MOESM3_ESM.docx]

Additional file 3: Table S1. cFos expression of the models (mean ± SEM)

|  | Models | | |
| --- | --- | --- | --- |
| Brain Regions | RU24969 (n=9) | 8-OH-DPAT (n=9) | MCPP (n=9) |
| LO±VO | 5.66±1.24 | 1.51±0.28 | 0.51±0.14 |
| MO | 2.27±0.6 | 0.86±0.1 | 0.58±0.12 |
| PRL | 1.72±0.28 | 0.93±0.11 | 0.83±0.18 |
| IL | 2.35±0.54 | 1.06±0.1 | 0.58±0.1 |
| Cg1 | 2.78±0.71 | 0.85±0.12 | 0.33±0.17 |
| Cg2 | 1.72±0.37 | 0.83±0.09 | 0.45±0.13 |
| CPu | 4.94±0.71 | 0.02±0.01 | 0.06±0.02 |
| AcbSh | 2.54±0.43 | 2.57±0.34 | 0.23±0.05 |
| AcbC | 2.77±0.64 | 0.12±0.03 | 0.16±0.02 |
| Hypothalamus | 3.61±0.34 | 1.95±0.17 | 1.01±0.24 |
| BSTLD | 9.55±1.21 | - | - |
| IPACL | 9.9±0.90 | - | - |
